# An analysis of the construct validity and responsiveness of the ICECAP-SCM capability wellbeing measure in a palliative care hospice setting

**DOI:** 10.1186/s12904-022-01012-4

**Published:** 2022-07-08

**Authors:** Gareth Myring, Paul Mark Mitchell, W. George Kernohan, Sonja McIlfatrick, Sarah Cudmore, Anne M. Finucane, Lisa Graham-Wisener, Alistair Hewison, Louise Jones, Joanne Jordan, Laurie McKibben, Deborah H. L. Muldrew, Shazia Zafar, Joanna Coast

**Affiliations:** 1 Health Economics Bristol, Population Health Sciences, Bristol Medical School, University of Bristol, Bristol, UK; 2 The National Institute for Health Research Applied Research Collaboration West (NIHR ARC West) at University Hospitals Bristol NHS Foundation Trust, Bristol, UK; 3 Institute of Nursing and Health Research, Ulster University, Newtownabbey, UK; 4 Division of Nursing, Queen Margaret University, Edinburgh, UK; 5 Centre for Clinical Brain Sciences, University of Edinburgh, Edinburgh, UK; 6 Clinical Psychology, University of Edinburgh, Edinburgh, UK; 7 Marie Curie Hospice, Edinburgh, UK; 8 School of Psychology, Queen’s University Belfast, Belfast, UK; 9 School of Nursing, Institute of Clinical Sciences, University of Birmingham, Birmingham, UK; 10 Marie Curie Palliative Care Research Department, University College London, London, UK; 11 School of Health, Wellbeing and Social Care, The Open University, Milton Keynes, UK

**Keywords:** Palliative care, Patient reported outcome measures, Economic evaluation, Psychometrics, Quality of life, Hospice care

## Abstract

**Background:**

For outcome measures to be useful in health and care decision-making, they need to have certain psychometric properties. The ICECAP-Supportive Care Measure (ICECAP-SCM), a seven attribute measure (1. *Choice*, 2. *Love and affection*, 3. *Physical suffering*, 4. *Emotional suffering*, 5. *Dignity*, 6. *Being supported*, 7. *Preparation*) developed for use in economic evaluation of end-of-life interventions, has face validity and is feasible to use. This study aimed to assess the construct validity and responsiveness of the ICECAP-SCM in hospice inpatient and outpatient settings.

**Methods:**

A secondary analysis of data collated from two studies, one focusing on palliative care day services and the other on constipation management, undertaken in the same national hospice organisation across three UK hospices, was conducted. Other quality of life and wellbeing outcome measures used were the EQ-5D-5L, McGill Quality of Life Questionnaire – Expanded (MQOL-E), Patient Health Questionnaire-2 (PHQ-2) and Palliative Outcomes Scale Symptom list (POS-S). The construct validity of the ICECAP-SCM was assessed, following hypotheses generation, by calculating correlations between: (i) its domains and the domains of other outcome measures, (ii) its summary score and the other measures’ domains, (iii) its summary score and the summary scores of the other measures. The responsiveness of the ICECAP-SCM was assessed using anchor-based methods to understand change over time. Statistical analysis consisted of Spearman and Pearson correlations for construct validity and paired t-tests for the responsiveness analysis.

**Results:**

Sixty-eight participants were included in the baseline analysis. Five strong correlations were found with ICECAP-SCM attributes and items on the other measures: four with the *Emotional suffering* attribute (*Anxiety/depression* on EQ-5D-5L, *Psychological* and *Burden* on MQOL-E and *Feeling down, depressed or hopeless* on PHQ-2), and one with *Physical suffering* (*Weakness or lack of energy* on POS-S). ICECAP-SCM attributes and scores were most strongly associated with the MQOL-E measure (0.73 correlation coefficient between summary scores). The responsiveness analysis (*n* = 36) showed the ICECAP-SCM score was responsive to change when anchored to changes on the MQOL-E over time (*p* < 0.05).

**Conclusions:**

This study provides initial evidence of construct validity and responsiveness of the ICECAP-SCM in hospice settings and suggests its potential for use in end-of-life care research.

**Supplementary Information:**

The online version contains supplementary material available at 10.1186/s12904-022-01012-4.

## Background

The cost of providing care for people in their last year of life is high, with one estimate of the average 2016 patient health care cost of four cancers in the final year of life ranging from £9579–£183,253 [1]. Patient benefit from healthcare services at the end of life is less well understood [2]. Information about life extending and/or life enhancing interventions are typically required in assessing the cost-effectiveness of healthcare interventions [3].

In estimating the cost-effectiveness of healthcare interventions, information on the quality of life that people experience is commonly collected. The EQ-5D health status measures (either with three or five levels) [4] are internationally recognised as suitable validated instruments to measure quality of life in health economic evaluation [5]. Having a single measure of quality of life, such as EQ-5D, allows for the generation of a combined metric that accounts for changes in the quality and quantity of life. The Quality Adjusted Life Year (QALY) enables consistent comparison of changes in the quality and quantity of life attributable to healthcare interventions in diverse patient groups. Such metrics are useful for decision-makers who need to allocate healthcare resources across healthcare systems.

The use of QALYs in healthcare decision-making, however, relies on a number of assumptions which are particularly challenging in the context of palliative care, where the appropriateness of the primary goal of QALY maximisation has been questioned [6–9]. One critique of using QALYs in palliative care is that the quality of life captured by QALYs does not accurately reflect what is important to people receiving palliative care [9].

The ICECAP - Supportive Care Measure (ICECAP-SCM) was developed to capture what matters most to those at the end of life for inclusion in health economic evaluation [10]. Value sets were developed, based on tasks conducted with representative samples of the UK general population to estimate the relative importance of each of the ICECAP-SCM’s seven attributes and levels [11]. Before the ICECAP-SCM can be widely used to inform healthcare decision-making, it is important to determine if it can measure outcomes reliably in the settings in which it is intended to be used. Palliative care services are generally under-resourced: in the UK, hospice care largely relies on funding from the voluntary sector. Understanding the relative costs and benefits of services, and interventions to improve care, is key to cost-effective planning of care for people approaching the end of life. The ICECAP-SCM, with its person-centred attributes, may provide more meaningful data relevant to palliative care than comparator measures suitable for general patient populations.

Previous use of the ICECAP-SCM has demonstrated its face validity and feasibility of use within a hospice care setting [12]; psychometric evaluation of its other measurement properties is yet to be undertaken. Construct validity explores the degree to which the relationship between two characteristics confirms previously anticipated theories [13]; one aspect of construct validity, convergent validity, assesses the degree of correlation between characteristics with similar constructs. The aim of this study was to examine whether the ICECAP-SCM measures the constructs it intends to (construct validity) and changes in those constructs over time (responsiveness) in hospice inpatient and outpatient environments [13].

## Methods

### Study design

The study was designed to determine construct validity and responsiveness and followed recommendations for analysis in the COSMIN (COnsensus-based Standards for the selection of health status Measurement INstruments) study design checklist for patient reported outcome measurement instruments [14].

### Sample

Data were collated from two studies conducted within the same setting, one a before-and-after cohort study examining the costs and outcomes of the use of palliative care day services (PCDS) [15] and the other exploring the use of an educational intervention for staff designed to improve the management of constipation in hospice patients [16, 17]. Both recruited from a national chain of hospices in the UK. All participants in both studies were over 18 years of age, were English speaking and provided written informed consent. In order to meet COSMIN criteria for adequate sample size, 30–49 participants were required for both construct and responsiveness analyses [14].

Patients in the constipation management study were eligible for recruitment by a healthcare provider if they had been admitted to an inpatient unit for symptom management which included constipation 6 months prior to intervention implementation and were assessed by the clinical team to be physically and psychologically able to participate. Patients were excluded if diagnosed with inflammatory bowel disease/any gastrointestinal disease of organic cause with associated constipation, constipation due to a lifelong/premorbid condition, or were actively dying.

Participants in the PCDS study were eligible for participant selection if they had a formal referral to PCDS. They were excluded if they were either: resident in a nursing/residential home, scored 40 or lower on the Australia-modified Karnofsky Performance Scale Index [18] or scored 3 or higher on the ECOG Scale of Performance Status [19] (both scales are clinical assessment tools completed as part of routine care on admission to PCDS, and can indicate inadequate performance and poor cognitive functioning), or a PCDS clinician determined they were too cognitively impaired to participate.

### Data collection

Participants were recruited between June 2017 and September 2018 in the PCDS study, and between October 2018 and June 2019 in the constipation management study. Outcome measures were completed either through face-to-face interviews (both studies) or independently by the patient and returned within 1 week (PCDS only). Ethical approval for the two studies was obtained from The Office of Research Ethics Committee Northern Ireland (reference: 18/NI/0074) and NHS Health Research Authority West Midlands - Solihull Research Ethics Committee (date: 9th May 2017, reference: 17/WM/0100). Approval was also obtained from Ulster University and the Research Governance Groups at each site.

### Outcome measures

Changes in outcome scores were expected where outcome measures were collected over time; a positive effect was expected because of the interventions, and a negative effect was expected due to worsening of the participants health.

#### ICECAP-supportive care measure (ICECAP-SCM)

The measure has seven attributes: *Choice*, *Love & affection*, *Physical suffering*, *Emotional suffering*, *Dignity*, *Being* s*upported*, and *Preparation*. Each attribute has four levels ranging from no capability to full capability on that attribute. Two slightly different interchangeable versions of the measure are available, one being more open about the end-of-life status of the patient [20], the other being less explicit, for example in the Preparation attribute (removing phrases related to funeral plans, and saying goodbye). The attributes of the ICECAP-SCM were identified during the instrument’s development by using in-depth interviews with people at various points on a trajectory towards the end of life, to identify what was most important to them.

The four levels of capability of each attribute are initially coded as a number representing the level of capability for that attribute, with 4 corresponding to the highest level of capability and 1 the lowest). This aids in describing the levels of capability at which individuals are situated. To generate a summary score, representing the sum of that individual’s capability, the findings from surveys of the general adult population about the relative importance people ascribe to the different attributes and levels of the ICECAP-SCM are used [11, 20]. These results from surveys are used to generate an index score value from zero (corresponding to no capability in all seven attributes) to one (corresponding to full capability in all seven attributes). The main analysis in this study used the interactions ICECAP-SCM value set, which factored in interactions between the seven attributes in the valuation [11].

#### EQ-5D-5L

The EQ-5D-5L is a health status measure [21]. Respondents rate their health by selecting one of five levels of severity (ranging from no problems to extreme problems) in five dimensions (*Mobility, Self-care, Usual activities, Pain/discomfort,* and *Anxiety/depression*). The EQ-5D-5L value set currently recommended by the National Institute for Health and Care Excellence (NICE) for use in the economic evaluation of health services [22] produces an index score ranging from − 0.594-1, anchored on a 0–1 (dead - perfect health) scale [23].

#### McGill quality of life questionnaire – expanded (MQOL-E)

The McGill Quality of Life Questionnaire – Expanded (MQOL-E) [24] contains 20-items over eight domains (*Physical, Psychological, Existential, Social, Burden, Environment, Cognition,* and *Healthcare*) and is designed to assess the quality of life of people with a life threatening illness. The mean of the eight domain scores is used to give an overall MQOL-E score between 0 and 10 (worst-best) [24].

#### Patient health questionnaire – 2 (PHQ-2)

The Patient Health Questionnaire – 2 (PHQ-2) [25] is a short two-item instrument designed for the assessment of depression. The respondent is asked how often over the last 2 weeks, ranging from nearly every day (3) to not at all (0), they have been bothered by the problems listed in each item *(Little interest or pleasure in doing things*, and *Feeling down, depressed or hopeless*). The overall score is produced by summing the scores of the two items, with a total score of 3 or greater indicating that a major depressive disorder is likely [25].

#### Palliative outcome scale – symptoms (POS-S)

The Palliative Outcome Scale – Symptoms (POS-S) is a symptom specific version of the Palliative Outcomes Scale, which was developed and validated for use in palliative care [26]. Respondents rate how severely they have been affected by ten symptoms over the previous week, ranging from overwhelmingly (4) to not at all (0). The ten symptoms listed are: *Pain, Shortness of breath, Weakness or lack of energy, Nausea, Vomiting, Poor appetite, Constipation, Mouth problems, Drowsiness,* and *Immobility*. An overall profile score is obtained by summing the scores for each of the 10 symptoms, ranging from 0 to 40 (best-worst).

### Analysis

Construct validity was assessed from baseline data in both studies. Responsiveness was assessed by comparing baseline and four-week follow-up data (collected in the PCDS study only). The convergent validity of the ICECAP-SCM was investigated in relation to the EQ-5D-5L, POS-S, PHQ-2, and MQOL-E, using data collected from both studies at baseline, by assessing: (i) the correlation between each of the domains of ICECAP-SCM and the domains of these other measures, (ii) correlation between the summary score of the ICECAP-SCM and these other measures’ domains, (iii) correlation between the ICECAP-SCM summary score and the summary scores of these other measures. It is important to note here that the measures refer to their constituent items/subscales in different ways (e.g. attributes for ICECAP-SCM, dimensions for EQ-5D-5L), and so in order to avoid confusion, from this point on the term ‘domains’ is used for all such elements.

It is good practice to generate a priori hypotheses, anticipating the expected relationships to be found, when validating outcome measures [27]. Hypotheses regarding the expected relationships between the ICECAP-SCM domains and those of the other measures were generated by three assessors (GM, PM, and JC). Each assessor independently assessed whether they thought the ICECAP-SCM domains would have a relationship with the domains of the other outcome measures. The results were collated and the raters met to discuss any disagreements between them and reach a consensus about expected relationships (see Additional file 1 for hypotheses generated).

Spearman rank correlation coefficients were calculated to assess the convergence of the domains of the five outcome measures. To assess the correlation between the final scores, Pearson’s correlation coefficients were used. Correlations were considered strong if coefficients were greater than 0.5 and moderate if between 0.3 and 0.5 [28]. As higher domain scores of the EQ-5D-5L, POS-S, and PHQ-2 domains and POS-S and PHQ-2 summary scores relate to worse health states, correlations with these and ICECAP-SCM were considered strong if coefficients were less than − 0.5 and moderate if between − 0.3 and − 0.5.

The responsiveness of the ICECAP-SCM score, that is whether the measure was sensitive to detect important or meaningful changes in the participants’ capability across time [13], was explored using outcome data provided by participants at both baseline and 4-week follow-up timepoints. The EQ-5D-5L and the MQOL-E were also completed at follow-up in the PCDS study, so both measures were considered for use as an “anchor”, the external criterion that changes in the ICECAP-SCM were based upon [29]. The appropriateness of the two measures to act as an anchor was tested based on their correlation with the ICECAP-SCM summary score from the baseline data. The follow-up data were split according to whether the score for the chosen anchor measure for different individuals improved or worsened over time, so that the mean change in the ICECAP-SCM score within the two groups could be calculated to assess whether increases in anchor measure scores corresponded with increases in ICECAP-SCM scores, and vice versa. Statistical analysis of paired t tests with 95% confidence intervals and effect size (Cohen’s d) were conducted to detect important or meaningful change for outcome measures collected over time and responsiveness analysis. Cohen’s d effect sizes are considered to be small if at least 0.2, medium if at least 0.5, and large if 0.8 and above [30].

The analyses described above were conducted using the ICECAP-SCM interaction scores as the main analysis [11]. Alternative scorings for the ICECAP-SCM using main effects scoring [11] and unweighted scoring (where all ICECAP-SCM domains and levels are given equal weight) were also analysed in a comparable way as the main analysis (see Additional file 2). Repeating the analyses in this way enabled comparison of the scoring methods to assess if one could be recommended over the other in this context. All analyses were carried out in Stata 15MP [31].

## Results

Fifty-six participants were recruited to the PCDS study, and outcome data collected using the ICECAP-SCM, EQ-5D-5L, MQOL-E, PHQ-2, and POS-S. Twelve patients were recruited to the constipation management in hospice patients study, and outcome data collected using the ICECAP-SCM and EQ-5D-5L. Outcome data were collected from 68 participants at baseline, 36 of whom also provided data at four-week follow-up. Fifty-seven participants provided outcome data through face-to-face interviews and 11 provided data independently by completing the measures at home and returning them within 1 week. The mean age of participants was 68.3 (standard deviation 11.9) across both studies with similar numbers of males and females recruited. All participants in the constipation management study and the majority in the PCDS study had a cancer diagnosis.

Mean baseline scores, for all participants who provided baseline data, as well as for the subsample who completed both baseline and follow-up data, are detailed in Table 1. While mean ICECAP-SCM summary scores at baseline for those who did and did not complete follow-up data were the same (0.66); the EQ-5D-5L scores differed slightly (0.46 and 0.52 respectively). A breakdown of individual responses to the ICECAP-SCM domains is provided in Additional file 3. Two patients did not complete the *Being prepared* domain of the ICECAP-SCM at baseline. There were no statistically significant changes or effect sizes reported for any of the measures collected at 4 weeks follow-up (see Table 1).

**Table 1:** Analysis sample characteristics

	Measure range	Mean baseline (***n*** = 68) value (SD)	Mean baseline (***n*** = 36) value of those with follow-up (SD)	Mean follow-up (***n*** = 36) value (SD)	Standardised difference (Cohen’s d)
Age		68.3^a^ (11.9)	68.8^a^ (9.6)	68.8 (9.6)	
Male		0.53	0.55	0.55	
ICECAP-SCM interaction summary score	0–1	0.66 (0.17)	0.66 (0.18)	0.65 (0.17)	0.01
EQ-5D-5L	−0.594−1	0.46 (0.32)	0.52 (0.29)	0.52 (0.30)	0.03
MQOL-E	0–10	6.54 (1.64)	6.50 (1.56)	6.50 (1.36)	0.00
PHQ-2	0–6	2.34 (1.87)			
POS-S	0–40	13.00 (6.13)			

*SD* standard deviation

^a^For a subsample of patients (*n* = 12) age at baseline was recorded in ranges. The midpoint of these ranges was used

### Construct validity – domain level

The convergence of the ICECAP-SCM domains and each of the other outcome domains is outlined in Table 2. In total, there were 25 moderate and five strong correlations found between ICECAP-SCM domains and those of the other measures. Twelve of these 30 correlations were with the *Emotional suffering* domain of the ICECAP-SCM. Ten of the 25 hypothesised associations were found as predicted. More associations were found with the MQOL-E and fewer with the EQ-5D-5L than predicted. Four of the five strong correlations were hypothesised relationships.

**Table 2:** Spearman’s correlation coefficients for ICECAP-SCM domains with EQ-5D-5L, POS-S, PHQ-2, and MQOL-E domains. *n* = 68

	ICECAP-SCM						
	Choice	Love and affection	Physical suffering	Emotional suffering	Dignity	Being supported	Preparation
**EQ-5D-5L**							
Mobility	− 0.03	0.08	**− 0.08**	0.12	− 0.13	0.00	− 0.14
Self-care	−0.12	0.13	**− 0.11**	− 0.03	− 0.23	0.05	− 0.02
Usual activities	0.00	0.18	**−0.16**	−0.18	− 0.15	0.11	− 0.17
Pain/discomfort	0.00	−0.08	**−0.24**	− 0.07	−0.28	− 0.19	−0.13
Anxiety/depression	**−0.15**	0.08	−0.06	**− 0.74**	− 0.41	−0.30	− 0.18
**MQOL-E**							
Physical	0.13	−0.20	**0.47**	0.36	0.31	0.07	0.29
Psychological	0.25	−0.13	0.11	**0.74**	0.28	0.23	0.07
Existential	0.13	0.14	0.31	**0.43**	0.14	0.33	**0.28**
Social	0.26	**0.07**	0.22	0.39	0.25	0.36	0.12
Burden	0.18	−0.05	0.12	0.52	0.18	0.15	0.17
Environment	0.12	0.25	0.09	0.17	0.07	0.28	0.27
Cognition	**0.05**	−0.01	0.22	0.47	0.32	0.39	0.17
Healthcare	0.14	0.01	−0.11	0.31	**−0.01**	**0.33**	0.23
**PHQ-2**							
Little interest or pleasure in doing things	−0.31	0.01	−0.22	**− 0.30**	− 0.27	− 0.38	− 0.13
Feeling down, depressed or hopeless	**−0.27**	0.09	−0.09	**− 0.59**	− 0.17	− 0.23	−0.06
**POS-S**							
Pain	0.06	0.11	**−0.31**	−0.26	− 0.21	0.00	0.09
Shortness of breath	−0.12	0.17	**−0.31**	−0.28	− 0.23	−0.13	− 0.44
Weakness or lack of energy	0.10	0.10	**−0.51**	−0.37	− 0.21	−0.04	− 0.31
Nausea	0.23	−0.12	**0.00**	−0.25	−0.28	− 0.18	0.10
Vomiting	0.11	0.01	**0.02**	−0.15	−0.25	− 0.30	−0.03
Poor appetite	0.27	0.07	−0.13	−0.08	− 0.25	0.01	0.04
Constipation	0.09	0.03	**−0.14**	−0.12	− 0.16	−0.10	0.17
Mouth problems	−0.09	0.01	**−0.15**	−0.31	− 0.25	− 0.21	− 0.13
Drowsiness	0.11	−0.13	0.03	−0.24	−0.19	− 0.22	−0.12
Immobility	−0.02	0.18	**0.01**	−0.18	−0.04	0.11	−0.04

Domains previously hypothesised to correlate are shown in bold. Attributes on ICECAP-SCM ordered in reverse to EQ-5D-5L, PHQ-2 and POS-S

The EQ-5D-5L domain, *Anxiety/depression,* correlated with three ICECAP-SCM domains. There were no other moderate correlations between EQ-5D-5L domains and ICECAP-SCM domains. The *Physical suffering* ICECAP-SCM domain did not show moderate correlations with any EQ-5D-5L domains. Excluding the *Environment* domain, which correlated close to moderate strength with *Being supported*, all MQOL-E domains recorded at least one correlation with an ICECAP-SCM domain, including the majority of MQOL-E domains being correlated with the *Emotional suffering* ICECAP-SCM domain. There was also a correlation between the *Emotional suffering* ICECAP-SCM domain and the PHQ-2 domains. There were three POS-S domains that correlated with *Physical suffering* and two that correlated with *Emotional suffering*, and *Preparation*, respectively.

### Construct validity – domain level

The ICECAP-SCM score was found to correlate well with all the MQOL-E domains, including strongly with four domains (*Physical, Existential*, *Cognition,* and *Social* – see Table 3*)*. The EQ-5D-5L *Anxiety/ depression* also had a strong correlation with the ICECAP-SCM score, as did *Pain/discomfort* with moderate strength. Both PHQ-2 domains were found to correlate moderately with the ICECAP-SCM score, as did the POS-S *Shortness of breath*, *Weakness or lack of energy*, and *Mouth problems* domains.

**Table 3:** Pearson’s correlation coefficients for the ICECAP-SCM score with the domains of the other measures. *n* = 68

	ICECAP-SCM score
**EQ-5D-5L**	
Mobility	−0.05
Self-care	−0.10
Usual activities	−0.17
Pain/discomfort	−0.31
Anxiety/depression	−0.56
**MQOL-E**	
Physical	0.53
Psychological	0.50
Existential	0.58
Social	0.53
Burden	0.41
Environment	0.24
Cognition	0.58
Healthcare	0.24
**PHQ-2**	
Little interest or pleasure in doing things	−0.46
Feeling down, depressed or hopeless	−0.41
**POS-S**	
Pain	−0.24
Shortness of breath	−0.44
Weakness or lack of energy	−0.48
Nausea	−0.21
Vomiting	−0.28
Poor appetite	−0.12
Constipation	−0.15
Mouth problems	−0.42
Drowsiness	−0.26
Immobility	−0.04

### Construct validity – summary score level

The ICECAP-SCM score also correlated with at least moderate strength with all scores of the other outcome measures, correlating strongly with MQOL-E, PHQ-2, and POS-S as well as correlating moderately with the EQ-5D-5L (Table 4).

**Table 4:** Pearson’s correlation coefficients for the ICECAP-SCM score with the scores of the other measures. *n* = 68

	ICECAP-SCM	EQ-5D-5L	MQOL-E	PHQ-2	POS-S
ICECAP-SCM	1.00				
EQ-5D-5L	0.30	1.00			
MQOL-E	0.73	0.18	1.00		
PHQ-2	−0.52	−0.21	−0.64	1.00	
POS-S	−0.52	−0.45	− 0.55	0.39	1.00

As higher scores of the PHQ-2 and POS-S relate to worse health states, correlations with the other measures in which higher scores relate to better states would be expected to be negative

### Responsiveness

MQOL-E was selected as an appropriate anchor measure to test the responsiveness to change of the ICECAP-SCM, given it had a stronger association compared to the EQ-5D-5L. Mean changes (standard deviation) in ICECAP-SCM and MQOL-E scores between baseline and four-week follow-up were − 0.01 (0.14) and 0.00 (1.23) respectively. Although no statistically significant change in either ICECAP-SCM or MQOL-E was reported at 4 weeks follow up in this sample, improvement in MQOL-E scores corresponded with an improvement in ICECAP-SCM scores and vice versa (see Table 5). There were statistically significant differences and small effect sizes recorded in scores between ICECAP-SCM scores at baseline and follow-up for those patients whose MQOL-E improved/worsened. Additional analyses in which the responsiveness analysis was repeated using alternative scoring of the ICECAP-SCM was also conducted (see Additional file 2).

**Table 5:** Responsiveness of the ICECAP-SCM score by MQOL-E anchor change groups. *n* = 36

	Number in group	Mean baseline (SD)	Mean follow-up (SD)	Mean change (SD)	Standardised difference (Cohen’s d)	***P*** value
ICECAP-SCM						
Improved MQOL-E	18	0.60 (0.16)	0.67 (0.17)	0.07 (0.11)	0.46	0.01*
Worsened MQOL-E	18	0.71 (0.18)	0.63 (0.18)	−0.08 (0.11)	0.44	0.01*

*SD* standard deviation

^*^Statistically significant difference at the 5% level using a paired t-test.

## Discussion

### Main findings

We present the findings of the first construct validity study of the ICECAP-SCM, a quality-of-life measure developed for use with people approaching the end of life. This study provides initial evidence of the construct validity and responsiveness to change of the ICECAP-SCM when used in hospice care settings. Construct validity was demonstrated in the high levels of correlation between the ICECAP-SCM and the other outcome measures designed to measure similar constructs, and the responsiveness to change of the ICECAP-SCM was found to be statistically significant.

There were strong correlations between the ICECAP-SCM *Emotional Suffering* domain, the EQ-5D-5L *Anxiety/depression* domain, the PHQ-2 domain *Feeling down, depressed or hopeless*, and the MQOL-E *Psychological* domain. The ICECAP-SCM was found to have strong correlations with the MQOL-E, at both the summary score and at the individual domain level. As the MQOL-E is designed to measure the impact of a life-threatening illness on general quality of life, this provides evidence to support the use of the ICECAP-SCM in this context. The ICECAP-SCM also correlated with the PHQ-2, a scale for measuring mental health. It is also notable that the ICECAP-SCM was not strongly associated with the EQ-5D-5L overall. The responsiveness of the ICECAP-SCM over time when anchored to the MQOL-E for better or worse outcomes over time was shown to result in statistically significant differences in capability over time.

### Strengths and limitations of the study

This study presents the first quantitative assessment of the construct validity and responsiveness of the ICECAP-SCM measure, adding to the evidence of its feasibility for use in hospice settings [12].

Recruiting research participants in end-of-life care settings can be difficult given the circumstances, particularly collecting data over a period of time in a setting in which the median days from referral to death is 48 days [32] thus leading to high levels of attrition occurring due to the deteriorating health or death of the participants. Although the study meets the COSMIN criteria for an ‘adequate’ sample size, further investigations with a larger sample may yield more robust evidence of the use of the ICECAP-SCM in this context. As this was the first use of the ICECAP-SCM, there were no available data to perform a sample size calculation. In addition, the research question on the validity of the ICECAP-SCM in hospice care was not the main focus of the studies where this data was collected, with sample size being driven by their primary research questions [15–17].

Our study also used two variations of the ICECAP-SCM, as the version that is more explicit about end of life approaching caused some distress for two participants in the PCDS study (the Preparation domain refers to “planning your funeral”). This led to a change in use of the ICECAP-SCM at the start of the PCDS study. Both, however, use the same scoring system; experience here suggests that the less explicit version of ICECAP-SCM may be more appropriate when patients are further from the end of life as well as when they are less aware of their prognosis.

### What this study adds

This study confirms that the ICECAP-SCM has the potential to be a reliable measure of the quality of life for people approaching the end of life. It provides evidence of construct validity and responsiveness to change of the ICECAP-SCM when used in hospice settings. The ICECAP-SCM is strongly associated with the MQOL-E, another recently developed measure for assessment of care at the end of life. There were also correlations found between the PHQ-2, and, to a lesser extent EQ-5D-5L and POS-S. This has important implications for choosing measures to include in the economic evaluation of end-of-life interventions, as it suggests that ICECAP-SCM may be sensitive to aspects of quality of life important to people near the end-of-life that are not captured by more established measurement tools used in economic analysis for decision-making. The responsiveness analysis suggests the ICECAP-SCM summary score is responsive to change when anchored on the MQOL-E measure of quality of life for people with a life-threatening illness [24], providing evidence of the ability of the ICECAP-SCM to effectively capture changes in a patients status over time due to deteriorating health or improvements as a result of an intervention.

These findings provide evidence of the validity of the ICECAP-SCM for use in hospice settings, however more research is needed in other end-of-life care settings such as at home in the community, in care homes, and in hospitals. This study demonstrates that, aside from the EQ-5D *Anxiety/depression* domain, there is little association between ICECAP-SCM and the EQ-5D. The EQ-5D is a measure focused on physical health functioning [33], and does not incorporate the aspects of quality of life captured by measures such as the ICECAP-SCM and MQOL-E. This could have significant implications when assessing the value of end of life care services if the EQ-5D is used in isolation. The complementarity of the EQ-5D-5L and ICECAP-SCM suggests that both should be included in future economic evaluation. While this analysis provides evidence for using the ICECAP-SCM with people who were mostly diagnosed with cancer, further investigation of the experience of patients on trajectories such as frailty and organ failure where the rate of changes in their conditions is different, would be beneficial [34].

We also recommend further consideration of the appropriate method to value ICECAP-SCM at the end of life, given standard approaches to valuation perform less well here (see Additional File 2). The responsiveness analysis suggests the ICECAP-SCM interaction summary score may be better at picking up changes in quality of life at the end of life compared to the main effects summary score. The interaction summary score also produced stronger correlations with the other measures tested here. Interactions between domains are not typically accounted for in the summary scores used in economic evaluation, so this study provides evidence in support of accounting for interactions. Interaction summary scores are more complex to understand and can produce unintuitive scores, yet this study suggests they may warrant closer attention in future. As people with advanced and progressive conditions deteriorate, some may adapt their preferences and these may differ from those values generated by the general public who are considering their end-of-life care in more abstract terms [35]; further valuation work with those at the end of life would inform the extent of these adjustments [36].

## Conclusion

This study provides initial evidence of the construct validity and responsiveness to change of the ICECAP-SCM when used to measure patient-centred capability in hospice settings. There is the potential to use the ICECAP-SCM as a more sensitive measure of the impact of palliative care in hospices, and other services in economic evaluation.

## Supplementary Information

**Additional file 1.** Hypothesised relationships

**Additional file 2.** Analysis of the ICECAP-SCM using alternative scoring mechanisms.

**Additional file 3.** Sample responses to the ICECAP-SCM.

## Data Availability

The data that support the findings of this study are available upon reasonable request from the corresponding author [PM]. The data are not publicly available due to them containing information that could compromise research participant privacy.
