# Supplementary material for: An analysis of the construct validity and responsiveness of the ICECAP-SCM capability wellbeing measure in a palliative care hospice setting
Source: BMC Palliat Care. 2022 Jul 8;21:121. doi: 10.1186/s12904-022-01012-4 (PMC9264696; doi:10.1186/s12904-022-01012-4)
Supplement: Supplementary file 1 — Additional file 1. Hypothesised relationships [file 12904_2022_1012_MOESM1_ESM.docx]

**Additional file 1: Hypothesised relationships**

The final consensus between the three raters on relationships between the ICECAP-SCM and other measure domains hypothesised to correlate with at least moderate strength.

*Table A1. Expected relationships reached through consensus between the three raters, marked X.*

|  | **ICECAP-SCM** | | | | | | |
| --- | --- | --- | --- | --- | --- | --- | --- |
|  | Choice | Love & friendship | Physical suffering | Emotional suffering | Dignity | Support | Preparation |
| **EQ-5D-5L** |  |  |  |  |  |  |  |
| Mobility |  |  | X |  |  |  |  |
| Self-care |  |  | X |  |  |  |  |
| Usual activities |  |  | X |  |  |  |  |
| Pain/discomfort |  |  | X |  |  |  |  |
| Anxiety/depression | X |  |  | X |  |  |  |
| **MQOL-E** |  |  |  |  |  |  |  |
| Physical |  |  | X |  |  |  |  |
| Psychological |  |  |  | X |  |  |  |
| Existential |  |  |  | X |  |  | X |
| Social |  | X |  |  |  |  |  |
| Burden |  |  |  |  |  |  |  |
| Environment |  |  |  |  |  |  |  |
| Cognition | X |  |  |  |  |  |  |
| Healthcare |  |  |  |  | X | X |  |
| **PHQ-2** |  |  |  |  |  |  |  |
| Little interest or pleasure in doing things |  |  |  | X |  |  |  |
| Feeling down, depressed or hopeless | X |  |  | X |  |  |  |
| **POS-S** |  |  |  |  |  |  |  |
| Pain |  |  | X |  |  |  |  |
| Shortness of breath |  |  | X |  |  |  |  |
| Weakness or lack of energy |  |  | X |  |  |  |  |
| Nausea |  |  | X |  |  |  |  |
| Vomiting |  |  | X |  |  |  |  |
| Poor appetite |  |  |  |  |  |  |  |
| Constipation |  |  | X |  |  |  |  |
| Mouth problems |  |  | X |  |  |  |  |
| Drowsiness |  |  |  |  |  |  |  |
| Immobility |  |  | X |  |  |  |  |
