# Supplementary material for: An analysis of the construct validity and responsiveness of the ICECAP-SCM capability wellbeing measure in a palliative care hospice setting
Source: BMC Palliat Care. 2022 Jul 8;21:121. doi: 10.1186/s12904-022-01012-4 (PMC9264696; doi:10.1186/s12904-022-01012-4)
Supplement: Supplementary file 2 — Additional file 2. Analysis of the ICECAP-SCM using alternative scoring mechanisms. [file 12904_2022_1012_MOESM2_ESM.docx]

**Additional file 2: Analysis of the ICECAP-SCM using alternative scoring mechanisms.**

Along with the interaction value set used for the primary analysis, a main effects value set is also available for producing summary scores, 0-1 (no capability-full capability), from the participant responses to the ICECAP-SCM questionnaire. Additionally, an unweighted ICECAP-SCM score, 7-28 (no capability-full capability), whereby all ICECAP-SCM domains and levels are treated equally and simply summed, can also be generated to assess the responsiveness of the levels of the domains, although this would not be recommended for use in the context of economic evaluation. A parallel analysis using these two alternative scores was also performed and is presented below for reference.

There was a notable difference in the mean summary scores produced by the interaction tariff (0.66 at baseline) compared with the main effects tariff (0.85 at baseline, see Table B1). As with the interaction summary scores, the unweighted and main effects summary scores correlated well with all the domains of the MQOL-E and PHQ-2 (Table B2). Overall, the ICECAP-SCM unweighted, main effects, and interaction summary scores correlated similarly with the domains of the other outcome measures. Again, the ICECAP-SCM unweighted and main effects summary scores correlated with the scores of the other outcome measures similarly to the interactions score, with the interactions score overall corelating with a slightly stronger degree with the other measures (Table B3). As with the interaction score, there was a statistically significant difference in scores between the ICECAP-SCM unweighted score at baseline and follow-up for patients whose MQOL-E scores improved/worsened, however this was not found with the main effects score (Table B5).

*Table B1. Analysis sample population ICECAP-SCM unweighted and main effects summary scores.*

|  | **Measure range** | **Mean baseline (n=68) value (SD)** | **Mean baseline (n=36) value of those with follow-up (SD)** | **Mean follow-up (n=36) value (SD)** | **Standardised difference (Cohen’s d)** |
| --- | --- | --- | --- | --- | --- |
| ICECAP-SCM unweighted score | 7-28 | 23.44 (2.74) | 23.42 (2.86) | 23.53 (2.65) | 0.04 |
| ICECAP-SCM main effects summary score | 0-1 | 0.85 (0.10) | 0.85 (0.11) | 0.86 (0.10) | 0.09 |

*Table B2. Pearson’s correlation coefficients for the unweighted and main effects scores with the other measures’ items. n=68*

|  | ICECAP-SCM unweighted score | ICECAP-SCM main effects summary score |
| --- | --- | --- |
| **EQ-5D-5L** |  |  |
| Mobility | -0.03 | -0.06 |
| Self-care | -0.06 | -0.06 |
| Usual activities | -0.14 | -0.04 |
| Pain/discomfort | -0.25 | -0.25 |
| Anxiety/depression | -0.53 | -0.51 |
| **MQOL-E** |  |  |
| Physical | 0.50 | 0.38 |
| Psychological | 0.48 | 0.40 |
| Existential | 0.56 | 0.50 |
| Social | 0.55 | 0.55 |
| Burden | 0.42 | 0.37 |
| Environment | 0.26 | 0.26 |
| Cognition | 0.58 | 0.54 |
| Healthcare | 0.25 | 0.21 |
| **PHQ-2** |  |  |
| Little interest or pleasure in doing things | -0.45 | -0.47 |
| Feeling down, depressed or hopeless | -0.41 | -0.34 |
| **POS-S** |  |  |
| Pain | -0.15 | -0.08 |
| Shortness of breath | -0.48 | -0.39 |
| Weakness or lack of energy | -0.47 | -0.35 |
| Nausea | -0.12 | -0.19 |
| Vomiting | -0.21 | -0.30 |
| Poor appetite | -0.05 | -0.07 |
| Constipation | -0.12 | -0.12 |
| Mouth problems | -0.41 | -0.37 |
| Drowsiness | -0.17 | -0.18 |
| Immobility | -0.03 | 0.02 |

*Table B3. Pearson’s correlation coefficients for unweighted and main effects scores with the other measures’ scores. n=68*

|  | **ICECAP-SCM unweighted score** | **ICECAP-SCM main effects summaryscore** | **EQ-5D-5L** | **EQ-5D-5L unweighted score** | **MQOL-E** | **PHQ-2** | **POS-S** |
| --- | --- | --- | --- | --- | --- | --- | --- |
| ICECAP-SCM unweighted score | 1.00 |  |  |  |  |  |  |
| ICECAP-SCM main effects summary score | 0.93 | 1.00 |  |  |  |  |  |
| EQ-5D-5L | 0.24 | 0.22 | 1.00 |  |  |  |  |
| EQ-5D-5L unweighted score | -0.27 | -0.24 | -0.95 | 1.00 |  |  |  |
| MQOL-E | 0.72 | 0.64 | 0.18 | -0.28 | 1.00 |  |  |
| PHQ-2 | -0.52 | -0.49 | -0.21 | 0.28 | -0.64 | 1.00 |  |
| POS-S | -0.44 | -0.38 | -0.45 | 0.53 | -0.55 | 0.39 | 1.00 |

*As higher scores of the EQ-5D-5L unweighted score, PHQ-2 and POS-S relate to worse health states, correlations with the other measures in which higher scores relate to higher health states would be expected to be negative.*

*Table B4. Mean scores at baseline and follow-up for the unweighted, main effects and MQOL-E scores. n=36*

| **Measure** | **Mean baseline (SD)** | **Mean follow-up (SD)** | **Mean change (SD)** |
| --- | --- | --- | --- |
| ICECAP-SCM unweighted score | 23.42 (2.86) | 23.53 (2.65) | 0.11 (2.15) |
| ICECAP-SCM main effects summary score | 0.85 (0.11) | 0.86 (0.10) | 0.01 (0.09) |
| MQOL-E | 6.50 (1.56) | 6.50 (1.36) | 0.00 (1.23) |

*Table B5. Responsiveness of the unweighted and main effects scores by MQOL-E anchor change groups. n=36*

|  | **Number in group** | **Mean baseline (SD)** | **Mean follow-up (SD)** | **Mean change (SD)** | **Standardised difference (Cohen’s d)** | **P value** |
| --- | --- | --- | --- | --- | --- | --- |
| ICECAP-SCM unweighted score |  |  |  |  |  |  |
| Improved MQOL-E | 18 | 22.50 (2.77) | 23.72 (2.82) | 1.22 (2.05) | 0.44 | 0.02* |
| Worsened MQOL-E | 18 | 24.33 (2.72) | 23.33 (2.52) | -1.00 (1.64) | 0.38 | 0.02* |
| ICECAP-SCM main effects summary score |  |  |  |  |  |  |
| Improved MQOL-E | 18 | 0.82 (0.12) | 0.86 (0.11) | 0.04 (0.09) | 0.33 | 0.09 |
| Worsened MQOL-E | 18 | 0.88 (0.10) | 0.86 (0.09) | -0.02 (0.07) | 0.21 | 0.27 |

** Statistically significant difference at the 5 % level using a paired t-test.*
