# Supplementary material for: An analysis of the construct validity and responsiveness of the ICECAP-SCM capability wellbeing measure in a palliative care hospice setting
Source: BMC Palliat Care. 2022 Jul 8;21:121. doi: 10.1186/s12904-022-01012-4 (PMC9264696; doi:10.1186/s12904-022-01012-4)
Supplement: Supplementary file 3 — Additional file 3. Sample responses to the ICECAP-SCM. [file 12904_2022_1012_MOESM3_ESM.docx]

**Additional file 3: Sample responses to the ICECAP-SCM.**

Responses of the analysis sample to the ICECAP-SCM questionnaire domains at both baseline and follow-up timepoints. Two patients had missing responses to the Preparation domain at baseline. Some domain, such as *Choice,* *Dignity,* and *Being supported* skew towards higher response level, possibly due to the hospice setting in which patients are provided constant care and support.

*Table C1. Response frequencies at baseline and follow-up to each of the ICECAP-SCM domain levels.*

| **Domains and Levels of ICECAP-SCM** | **Baseline (n=68) frequency (%)** | **Follow-up (n=38) frequency (%)** |
| --- | --- | --- |
| Choice 1  2  3  4 | 2 (3%)  0 (0%)  3 (4 %)  63 (93%) | 1 (3%)  1 (3%)  3 (8%)  33 (87%) |
| Love and affection  1  2  3  4 | 0 (0%)  3 (4%)  7 (10%)  58 (85%) | 0 (0%)  1 (3%)  1 (3%)  36 (95%) |
| Physical suffering  1  2  3  4 | 23 (34%)  21 (31%)  14 (21%)  10 (15%) | 16 (42%)  11 (29%)  9 (24%)  2 (5%) |
| Emotional suffering  1  2  3  4 | 14 (21%)  11 (16%)  21 (31%)  12 (32%) | 8 (21%)  11 (29%)  10 (26%)  9 (24%) |
| Dignity  1  2  3  4 | 0 (0%)  4 (6%)  3 (4%)  61 (90%) | 0 (0%)  0 (0%)  3 (8%)  35 (92%) |
| Being supported  1  2  3  4 | 0 (0%)  6 (9%)  4 (6%)  58 (85%) | 1 (3%)  1 (3%)  3 (8%)  33 (87%) |
| Preparation  1  2  3  4 | 10 (15%)  8 (12%)  10 (15%)  38 (56%) | 5 (13%) 0 (0%)  4 (11%)  29 (76%) |
